# Parental perspectives on access to paediatric oncology clinical trials and treatment options: A cross-sectional survey

**DOI:** 10.6026/9732063002001959

**Published:** 2024-12-31

**Authors:** Yuvaraj Perumal, Yogesh S., Roshni S., Saranya R., Nithyashree Sathiyamurthy, Gajalakshmi, Yagvalkya Sharma, Mohana Kanthamneni

**Affiliations:** 1 Department of Neuro ICU, People Tree Hospitals, Tumkur Road, Goraguntepalya, Bengaluru, Karnataka, India; 2 Institute of Internal Medicine, Madras Medical College & Rajiv Gandhi Government General Hospital, MHPE Scholar, Sri Balaji Vidyapeeth University, Pondicherry; 3 Department of Pediatrics, Madras Medical College, Chennai, India; 4 Department of Community Medicine, Madha Medical College and Research Institute, Chennai, India; 5 Institute of Internal Medicine, Madras Medical College, Chennai, Tamil Nadu, India; 6 Department of Community, Veer Chandra Singh Garhwali Government Institute of Medical Science and Research, Srinagar, Pauri Garhwal, Uttarakhand, India; 7 Department of Research, Kalp Research Work, Mathura, Uttar Pradesh, India; 8 Department of Pediatrics, Fernandez Hospital Hyderabad, Telangana, India

**Keywords:** Paediatric oncology, clinical trials, parental perspectives, access to treatment, decision-making, barriers, cross-sectional survey

## Abstract

Parents of children with cancer often face complex decisions about clinical trial participation and treatment options. This cross-sectional survey of 100 parents of pediatric oncology patients explored their perspectives on accessing clinical trials, focusing on influencing factors and perceived barriers. While most parents expressed interest in clinical trial participation, common obstacles included inadequate information, geographic constraints and financial challenges. Many parents emphasized the need for improved communication with healthcare providers to better understand trial risks and benefits. These findings suggest that addressing informational and logistical barriers can enhance access to clinical trials and support informed decision-making among parents of pediatric oncology patients.

## Background:

Paediatric oncology is a fast-moving field where treatment innovation features prominently in the utilization of clinical trials. Clinical studies forward treatment as novel therapies are viable avenues for children afflicted with cancers that might not readily respond to traditional treatment options [1]. Clinical trials introduce new drugs, targeted therapies and immunotherapies, which are avenues for improved survival rates as well as minimized long-term effects of the side sequence associated with conventional cancer therapies. This development is quite significant because despite the happening of so many good developments in the field of medicine, cancer continues to be among the major causes of children's death; hence, easily accessible and effective treatment methods are urgently needed [2]. Parents have to navigate something of a minefield in terms of eligibility criteria, informed consent and practical requirements, not to mention the geographic disparities that may mean that specialized paediatric oncology centres are far removed from rural populations [3]. Financial, temporal and emotional stresses also add considerable burdens on parents when pursuing trials for their child. Studies have shown most parents who take their children into clinical trials are unaware of such trials that exist and are not well-informed of the processes of enrolling their child. The state of no knowledge and little understanding disarms their decision-making and limits them to include a lot of what may be available [4]. At the same time, they tend to be more optimistic about experimental treatment when a patient trusts the healthcare provider, when new therapies are perceived to be safe and effective and the results of the trial are transparent [5]. A parent may avoid or is not willing to enter such a trial due to the risk of developing complications or side effects not documented anywhere or the lack of information on the follow-ups after some time [6]. These concerns are often compounded by the emotional stress of managing their child's illness and the burden of having to make life-changing decisions on behalf of their child. Effective communication, comprehensive education and continuous support from healthcare providers are critical in helping parents make informed choices regarding their child's participation in clinical trials [7, 8]. Therefore, it is of interest to would concentrate on understanding the perceptions of parents toward the paediatric oncology clinical trials, including the related access barriers, worries involved in the decision-making process and information needs. Such an investigation may help find insights necessary to inform policies and practices that improve access to such clinical trials, support the process of parental decision-making and improve a more comprehensive care experience for families dealing with paediatric oncology.

## Methodology:

This cross-sectional survey sought to investigate the views of parents regarding access to paediatric oncology clinical trials and treatments-which influences decision-making and what prevents parents from participating. To date, this study has a sample size of 100 parents whose children were diagnosed with cancer; these participants are drawn from multiple treatment centres in the United States. All parents of children who are receiving treatment for cancer were included in this survey, irrespective of whether their child was involved in a clinical trial. Screening was conducted with the parents of children who had been discharged from therapy or were now only on palliative care to rule out irrelevant participants and make sure the study was of relevance to the active treatment and trial eligibility. A structured, self-administered questionnaire was developed after a literature review in consultation with oncology experts. The survey had 30 questions under three sections: demographic information, awareness and understanding of clinical trials and perceived barriers to access. Demographic questions collected information regarding the child's age, the type of cancer and the geographical location of the treatment centre. Information regarding the socioeconomic status and level of education of the parents was also collected. Awareness questions for parents involved assessment of their awareness with respect to clinical trials, sources of information and perceptions of risk versus benefit. The barriers section consisted of multiple-choice and open-ended questions in which parents could elaborate difficulties in detail, such as coordination, information and finance. Data collection took place over three months. The paper or electronic survey could be completed on the preferred medium of the parents, as recruitment took place in routine clinic visits and was fully explained by the study staff regarding the purpose and procedures of the study with parental consent prior to agreeing to participate. Anonymity was assured so that honest responses were achieved and reminder notifications were included to maximise response rates half-way through the period of data collection. Data were analyzed using SPSS (Version 25.0). Estimation of the demographic and survey data involved descriptive statistics, including mean, standard deviation and frequency distributions. Chi-square and t-tests were used to assess whether demographic factor variability between groups had an impact on awareness and perceived barriers to treatment; the level of statistical significance in this study was set at a value of p < 0.05. Institutional Review Board Ethical Approval the Institutional Review Board Ethical Approval was taken and the participation in this study was entirely voluntary. Confidentiality and anonymity were strictly maintained and all data were stored securely. It was informed to the parents that they could withdraw at any stage of the study without affecting any treatment or clinical care for their child.

## Questionnaire:

The questionnaire had parts of survey that included structured sections for assessing demographic characteristics, knowledge of clinical trials, experience in access and perceptions of the available treatment options. There were also open-ended questions placed for parents to comment about their experiences, concerns and suggestions about how to improve access to the trials.

## The Table 1 below shows the Summary of the Questionnaire Structure:

The questionnaire probes parental views on pediatric oncology clinical trials by questioning the most relevant areas of awareness, decision-making factors, barriers to participation and overall experiences. It looks at parents' knowledge of trials and treatment options, factors influencing decisions and challenges such as accessibility and safety concerns. In addition, it gathers feedback on communication with healthcare providers and satisfaction with the information provided while seeking suggestions to enhance access, support and communication. This is a holistic approach intended to identify gaps and opportunities in improving the trial process and supporting systems for families.

## Results:

Table 2 presents an overview of the participant demographics, including the age of the child, type of cancer and geographic distribution. Table 3 highlights the level of awareness parents had about clinical trial options for paediatric oncology, indicating varying degrees of knowledge. Table 4 present the barriers parents reported facing when seeking clinical trials for their children, with logistical and financial challenges as prominent factors. Table 5 reflects parents' trust in experimental treatments compared to standard treatments, showing varied levels of trust in novel therapies. Table 6 indicates the types of information parents seek to make informed decisions about clinical trial participation, with a preference for clear, comprehensive explanations. Table 7 summarizes parents' satisfaction with the support provided by healthcare professionals, showing varied satisfaction levels. Table 8 shows the distance families need to travel to reach a clinical trial centre, highlighting logistical challenges for rural families. Table 9 presents parents' willingness to enrol their child in a clinical trial if recommended by their physician. Table 10 lists where parents obtained information about clinical trials, emphasizing reliance on healthcare providers and online resources. Table 11 highlights themes from open-ended responses, such as the need for better information and emotional support.

## Discussion:

This study provides perceptions on access to paediatric oncology clinical trials and treatment among parents, identifying barriers that may influence their decision and choices. As such, many parents found that the most interested group was keen on participating in clinical trials, but there are huge barriers like lack of information, distances and financial issues that hinder access and affect their major decisions [9]. These findings emphasize the need for greater communication, support mechanisms and resources in helping families make informed decisions about entering clinical trials. The findings of this study indicate that parents do have some knowledge of the benefits of clinical trials but perceive insufficient information to exercise a fully-informed decision-making process. Other researchers have cited the unavailability of accessible information in paediatric oncology as one of the main barriers to the entry of children into clinical trials. Parents would find it challenging to go through the scientific and procedural nature of trials [10]. The gap requires health care providers to provide transparent information. More information on the trial, rather explained in simpler words and customized education materials can help parents make informed decisions [11, 12]. Parents' confidence in determining what is in the best interest of their child may be bolstered by the assurance that they understand the benefits and drawbacks associated with participating in a clinical trial. Regional area was identified as the biggest limitation, where parents whose homes are located further from the clinical trial site face the greatest difficulty in reaching the facility. Indeed, this finding falls in line with studies that cite geographic constraints as one limitation to participating in paediatric oncology trials due to the distance to centres that provide the specialized treatment often being what prevents access to them for some of the families residing outside of the major urban areas [13]. Indeed, many of the families find it challenging traveling to these trial sites as it incurs time away from their work and additional financial cost that might not be very feasible. This can be overcome by offering Decentralized Clinical trials or expanding the number of trial sites to more areas so that the travel requirements are minimized to the family members. The latest developments in telemedicine and mobile health technologies can also be used to increase access because some aspects of trial participation may be done remotely, thereby reducing the requirements to make frequent visits to the clinics [14]. Economic burden was the most significant barrier encountered. This is evidenced by the many parents who expressed worry about the monetary cost of travel and accommodations and other related trial expenditures. This finding is comparable with other studies and research that pointed out the economic burden borne by the families affected when participating in clinical trials [15]. To assist in reducing these barriers to trial participation, healthcare institutions and involved trial sponsors may consider providing financial aid or travel support to families. In addition, increased support to families with more financial counselling in addition to support organization referrals may be instrumental in decreasing some of the financial stress imposed by participating in clinical trials [16]. The parents indicated a great need for better communication with the provider as well as the fact that providers made such a crucial difference in deciding on clinical trials. The communication between providers and families is essential because it will ensure parents know the different trial options, eligibility criteria and potential impacts of participation. It has been reported that when clinical trials are explained in a compassionate manner, parents will be comfortable and confident about the decision [17]. The research findings would support the necessity of having a proper framework for communication in the clinics that handle oncology and parents should also be adequately supported and informed. Providers could benefit from further training so that they could communicate more effectively with families facing tough decisions in paediatric oncology [18]. The study indicates the following areas on which healthcare providers and policy makers could work to enhance accessibility of clinical trials in paediatric oncology [19]. Support to these can be provided through education for the patient; reduction of financial and geospatial barriers and promoting the providers to communicate with parents. On the policy side, increasing regional trial centres or allowing for decentralization would reduce the geospatial barrier to regional locations of residence and financial assistance policies that would cover the cost of testing would serve to reduce the economic burden on families. Such interventions would, therefore, ensure that families of all socioeconomic and geographical locations get an equal opportunity to access potentially life-saving clinical trials. Despite the insights this study might provide, there are nonetheless limitations that merit attention. The sample size was small, so the findings may not apply to the entire cross-section of parents of paediatric oncology patients. Only a snapshot of views would be captured by the cross-sectional study design and nothing is accounted for about how things change over time or as options in treatment evolve. Future research could include longitudinal assessments of parental perspectives. More research on larger and more diverse samples may be helpful for better understanding the variations in parental attitude in different demographic and socioeconomic groups [20]. The findings from this pilot study suggest that a goal of future informed consent interventions should be to improve parents' understanding of the research aspects of treatment [21]. P1Ts are essential to the development of novel therapies for childhood cancers; however, they raise ethical concerns about balancing the need for the trials with maintaining the well-being of the participating children and their families. P1T experiences were found to be primarily positive in this qualitative study, although P1T processes and procedures posed some challenges [22].

## Conclusion:

This challenge faced by parents whose children are diagnosed with cancers is multi-dimensional, including the impediments that involve coordination, financial considerations, informational gaps and concerns related to the risks of treatment. Facilitating better communication, more adequate support systems and appropriately targeted resources may help ensure informed decisions from parents while opening up access to trials for every child. In support of this, supportive infrastructure and constant and transparent information delivery are some of the elements that can be understood to be competent in emancipating parents to make an informed decision regarding the clinical trial options available to the child.

## Figures and Tables

**Table 1:** Summary of the questionnaire

**Section**	**Focus Area**	**Details Included**
Demographics	Background information	Age of child, type of cancer, treatment history, geographic location
Awareness of Clinical Trials	Knowledge of clinical trial options	How informed are you about clinical trials available for paediatric oncology?
Perceived Barriers to Access	Challenges in accessing trials	What challenges have you encountered in seeking clinical trials for your child?
Trust in Experimental Treatments	Attitudes toward new therapies	How much do you trust experimental treatments as compared to standard options?
Information Needs	Preferences for educational resources	What types of information would you find helpful when considering a clinical trial?
Support Systems	Experience with healthcare support	Are you satisfied with the support provided by healthcare providers regarding trial options?
Open-Ended Responses	Additional insights from parents	Please share any specific experiences or recommendations regarding trial access in paediatric oncology.

**Table 2:** Demographic characteristics of participants

**Variable**	**Percentage (%)**
Age of Child (0-5)	25
Age of Child (6-10)	40
Age of Child (11-15)	30
Age of Child (16+)	5
Leukemia	35
Brain Tumor	25
Other Cancers	40
Urban Location	55
Rural Location	45

**Table 3:** Awareness of clinical trials

**Awareness Level**	**Percentage (%)**
Very Aware	20
Somewhat Aware	40
Not Aware	40

**Table 4:** Perceived barriers to clinical trial access

**Barrier**	**Percentage (%)**
Logistical Difficulties	55
Financial Barriers	50
Lack of Information	45
Emotional Distress	30

**Table 5:** Trust in experimental treatments

**Trust Level**	**Percentage (%)**
Very Trusting	25
Somewhat Trusting	50
Not Trusting	25

**Table 6:** Information needs for decision-making

**Information Type**	**Percentage (%)**
Detailed Trial Info	65
Success Rates	50
Long-Term Effects	45
Risks and Side Effects	60

**Table 7:** Satisfaction with healthcare support

**Satisfaction Level**	**Percentage (%)**
Very Satisfied	30
Satisfied	40
Neutral	20
Dissatisfied	10

**Table 8:** Distance to treatment centres

**Distance Category**	**Percentage (%)**
Less than 50 miles	40
50-100 miles	35
More than 100 miles	25

**Table 9:** Parental willingness to enrol in trials

**Willingness Level**	**Percentage (%)**
Very Willing	40
Somewhat Willing	45
Not Willing	15

**Table 10:** Sources of clinical trial information

**Information Source**	**Percentage (%)**
Healthcare Provider	60
Internet	50
Support Groups	35
Media	20

**Table 11:** Common themes from open-ended responses

**Theme**	**Percentage of Responses (%)**
Need for Emotional Support	55
Better Trial Information	40
Financial Assistance	35
